# Heart Rate Methods Can Be Valid for Estimating Intensity Spectrums of Oxygen Uptake in Field Exercise

**DOI:** 10.3389/fphys.2021.687566

**Published:** 2021-07-06

**Authors:** Jane Salier Eriksson, Karin S. E. Olsson, Hans Rosdahl, Peter Schantz

**Affiliations:** ^1^The Research Unit for Movement, Health and Environment, Department of Physical Activity and Health, The Swedish School of Sport and Health Sciences, GIH, Stockholm, Sweden; ^2^The Research Unit for Movement, Health and Environment, Department of Physiology, Nutrition and Biomechanics, The Swedish School of Sport and Health Sciences, GIH, Stockholm, Sweden

**Keywords:** heart rate method, oxygen uptake, heart rate-oxygen uptake relationship, metabolic measurements, cycling, cycle commuting

## Abstract

**Purpose:**

Quantifying intensities of physical activities through measuring oxygen uptake (V̇O_2_) is of importance for understanding the relation between human movement, health and performance. This can in principle be estimated by the heart rate (HR) method, based on the linear relationship between HR and V̇O_2_ established in the laboratory. It needs, however, to be explored whether HR methods, based on HR-V̇O_2_ relationships determined in the laboratory, are valid for estimating spectrums of V̇O_2_ in field exercise. We hereby initiate such studies, and use cycle commuting as the form of exercise.

**Methods:**

Ten male and ten female commuter cyclists underwent measurements of HR and V̇O_2_ while performing ergometer cycling in a laboratory and a normal cycle commute in the metropolitan area of Stockholm County, Sweden. Two models of individual HR-V̇O_2_ relationships were established in the laboratory through linear regression equations. Model 1 included three submaximal work rates, whereas model 2 also involved a maximal work rate. The HR-V̇O_2_ regression equations of the two models were then used to estimate V̇O_2_ at six positions of field HR: five means of quintiles and the mean of the whole commute. The estimations obtained were for both models compared with the measured V̇O_2_.

**Results:**

The measured quintile range during commuting cycling was about 45–80% of V̇O_2_max. Overall, there was a high resemblance between the estimated and measured V̇O_2_, without any significant absolute differences in either males or females (range of all differences: −0.03–0.20 L⋅min^–1^). Simultaneously, rather large individual differences were noted.

**Conclusion:**

The present HR methods are valid at group level for estimating V̇O_2_ of cycle commuting characterized by relatively wide spectrums of exercise intensities. To further the understanding of the external validity of the HR method, there is a need for studying other forms of field exercises.

## Introduction

Quantification of physical activity intensities is of importance for understanding the relation between human movement, health and performance. Preferably, they shall be based on both absolute and relative levels of oxygen uptake (V̇O_2_). Measurements of V̇O_2_ with mobile metabolic systems in such conditions are, however, difficult both on a large scale and for technical reasons (Salier Eriksson et al., 2012; Schantz et al., 2018). For both research, health promotion and educational purposes, it would therefore be helpful if the heart rate (HR) method for estimating V̇O_2_ is valid in field conditions.

The HR method is based on the linear relationship between HR and V̇O_2_ with increased work rates under steady state conditions (Boothby, 1915; Krogh and Lindhard, 1917; Hohwü Christensen, 1931; Berggren and Hohwü Christensen, 1950). When an individual HR-V̇O_2_ relationship has been established under such controlled conditions, V̇O_2_ can in principle be estimated in the field by HR measurement for a wide range of intensities [with individual exceptions at very low and very high intensity levels (Booyens and Hervey, 1960; Davies, 1968; Achten and Jeukendrup, 2003)].

However, as Berggren and Hohwü Christensen (1950) stated, the HR method must be used “with great care” since the HR “can vary independent of metabolic rate.” The ingredients in the Fick’s principle [V̇O_2_ = HR × SV × (a-v̄ O_2_-difference)] indicate the potential variability in this respect. Extensive investigations of the HR-V̇O_2_ relationship in various activities show that it can be affected by the character of the physical work, such as the amount of muscle mass engaged (Stenberg et al., 1967; Vokac et al., 1975; Eston and Brodie, 1986), the type of muscle contractions (e.g., dynamic vs. static) (Maas et al., 1989) and the position of the body (e.g., sitting vs. supine) (Bevegård et al., 1966; Stenberg et al., 1967). Furthermore, longer exercise durations and dehydration can give rise to a drift in HR (Ekelund, 1967; Coyle and Gonzalez-Alonso, 2001; Achten and Jeukendrup, 2003), which needs to be taken into consideration by the HR method in case it is not accompanied by a similar drift in V̇O_2_ (Zuccarelli et al., 2018). Differences of temperature and humidity between controlled laboratory conditions and the ambient conditions may also affect HR (Galloway and Maughan, 1997; Achten and Jeukendrup, 2003), as well as stressful situations (Carroll et al., 2009; Hahad et al., 2019).

Due to the above mentioned influencing factors related to the HR-V̇O_2_ relationship, methodological evaluations are of importance for developing this method. Given this background we have, as an initial step, investigated the reproducibility of the HR method in the laboratory. Based on measurements of HR and V̇O_2_ while performing submaximal and maximal work rates of ergometer cycling, we were able to demonstrate a high reproducibility at the group level with male and female active commuters (Schantz et al., 2019a,b).

The validity of HR monitoring as a method for estimating V̇O_2_ or energy expenditure in both standardized laboratory and free-living physical activities has been studied in various ways during the latter part of the 1900s (e.g., Rodahl et al., 1974; Spurr et al., 1988; Luke et al., 1997; Eston et al., 1998; Strath et al., 2000). Despite these examinations, laboratory measured HR-V̇O_2_ relationships that are applied to estimate intensity spectrums of V̇O_2_ have, to our knowledge, not previously been evaluated with a valid method in any form of field exercise. This lack of knowledge has determined our next and essential step in our methodological development of the HR method.

One form of physical activity that would be particularly valuable for this validation is cycle commuting. This is since it has a potential to be incorporated into a daily life routine and contribute to population health through a reduction of morbidity (e.g., Hu et al., 2003; Hu et al., 2007; Pucher et al., 2010) and risk for premature mortality (Andersen et al., 2000; Matthews et al., 2007). At the same time, not much research is available concerning the physical activity bases for such health outcomes (Stigell and Schantz, 2015; Schantz, 2017; Schantz et al., 2020).

Given this background, the aim of this study was to explore whether HR methods, based on HR-V̇O_2_ relationships established in the laboratory, are valid for estimating intensity spectrums of V̇O_2_ in field exercise, with special reference to cycle commuting. For that purpose, ten male and ten female habitual commuter cyclists underwent measurements of HR and V̇O_2_ while performing ergometer cycling in a laboratory and their normal cycle commutes in the metropolitan area of Stockholm County, Sweden. Two models of HR-V̇O_2_ relationships were individually established in the laboratory through linear regression equations. Model 1 included three submaximal work rates, whereas model 2 included also a maximal work rate. Potentially, the inclusion of a maximal work rate could stabilize the HR-V̇O_2_ relationship. The two models’ HR-V̇O_2_ regression equations were then used to estimate V̇O_2_ at six individual positions of HR in field: five means of quintiles and the mean of the whole cycle commute. The estimated and measured levels of V̇O_2_ at the six HR positions were then compared for both models of HR methods.

## Materials and Equipment

### Laboratory Tests

#### Stationary Metabolic System

A stationary metabolic system (SMS), the Oxycon Pro^®^, (Carefusion GmbH, Hoechberg, Germany) was used in the mixing chamber mode for all metabolic measurements in the laboratory. The software used was JLAB 4.53. In this system oxygen is measured by a paramagnetic gas analyzer and carbon dioxide by an infra-red gas analyzer. Expired air is sampled continuously from the mixing chamber through nafion tubing on the outside of the equipment that connects to another nafion tubing inside the equipment terminating at the analyzer inlets. Ventilation is measured through a digital volume transducer which is attached to the outlet of the mixing chamber. The equipment was switched on 30 min before data collection and calibrated immediately before and after each test using the built-in automated procedures in accordance with the manufacturer’s recommendations. A high precision gas of 15.00% O_2_, and 6.00% CO_2_ (accuracy: O_2_ ± 0.04% and CO_2_ ± 0.1%; Air Liquid AB, Kungsängen, Sweden) was used for calibration. A face mask with non-rebreathing air inlet valves (Combitox, Dräger Safety, Lübeck, Germany) was used. The mask was carefully fitted on each subject and controlled for air leakage immediately prior to the measurements. A tube (inner diameter of 35 mm) attached to the mask led the expired air into the mixing chamber. All measured metabolic variables were saved in averages of 15 s.

#### Heart Rate Monitor

HR was recorded using a Polar Wearlink 31 transmitter (Polar Electro Oy, Kempele, Finland). The HR values were saved in averages of 15 s and stored in the metabolic system.

#### Cycle Ergometer

A manually braked pendulum cycle ergometer (828E Monark Exercise AB, Vansbro, Sweden) was used for the cycle exercise in the laboratory. Before each test, the scale was zeroed while each subject sat on the saddle with his or her feet resting on the area between the pedals. The saddle height was adjusted so that the participant’s knees were slightly flexed when the feet were on the pedals in their lowest position. Throughout the submaximal ergometer cycling, the participants sat in an upright position with their hands laying on the handlebars. A digital metronome (DM70 Seiko S-Yard Co. Ltd., Tokyo, Japan) was used to help the participants maintain correct cycling cadence. The body posture and the work rates were controlled every minute by checking the cadence and the braking force as indicated on the pendulum scale.

### Field Tests

#### Mobile Metabolic System

A mobile metabolic system (MMS) (Oxycon Mobile, version 5.10. CareFusion GmbH, Hoechberg, Germany) was used for the field tests. Gas exchange and ventilation variables were measured breath by breath. This version of the MMS has been carefully validated and is described in detail elsewhere (Rosdahl et al., 2010; Salier Eriksson et al., 2012; Schantz et al., 2018). Preparations and calibration procedures were undertaken in the same manner as described for the SMS. Note, however, that the calibrations were always performed in the same environmental settings as the different measurement conditions. The same face masks were used as during the laboratory tests. The metabolic values were transformed and saved in averages of 15 s.

#### Heart Rate Monitor

HR was recorded and saved in the same manner as described for the laboratory tests. As a safety measure, HR values were also stored in averages of 15 s in the Polar Electro S610i HR monitor watch (Polar Electro Oy, Kempele, Finland). The HR values were always used from the MMS, except for a total of three short periods, one in each of three participants, when values were missing due to technical problems with the MMS. In these cases, the HR monitor watch’s values replaced the missing HR values. To confirm that this was viable, all individuals’ watch HR values were compared against the corresponding MMS HR values. No significant differences were found between the two methods. The lengths of time in which the watch’s HR values replaced the missing MMS HR values for the three participants were 375, 105, and 60 s, respectively.

## Methods

This study is part of a greater multidisciplinary research project, Physically Active Commuting in Greater Stockholm (PACS), at the Swedish School of Sport and Health Sciences, GIH, in Stockholm, Sweden.

### Participants

The process of recruiting participants for the entire research project (PACS) was divided into several steps and is previously described in detail in Stigell and Schantz (2015). The overall inclusion criteria were: being at least 20 years old, living in the county of Stockholm (excluding the municipality of Norrtälje) and walking or cycling the whole way, any distance, to one’s work or place of study at least once a year. A questionnaire was used to obtain descriptive information for selecting participants, including e.g., sex, age, commuting mode, commuting frequency per week for each month of the year as well as commuting time and distance. The commuting distance was measured on routes drawn in maps by each respondent (Schantz and Stigell, 2009). The present participant sample was selected from the cyclist category, i.e., those subjects who only cycled to work (no electrically assisted or semi-recumbent bicycles were included). Other specific criteria for the present study were that the participants had ages and route distances close to the overall median values of the male and female cyclists, respectively (Stigell and Schantz, 2015). They also rated their daily professional jobs as light or very light physically. Based on this information, 10 male and 10 female habitual commuter cyclists who fulfilled the criteria were selected for participation (Table 1). All had answered a health declaration and certified themselves to be healthy for participation (individuals with high blood pressure or on medication that could affect normal HR were excluded). Prior to participation, subjects also signed a consent of participation after being informed about the physical tests and their rights as participants. Approval to conduct this study was obtained from the Ethics Committee North of the Karolinska Institute at the Karolinska Hospital (Dnr 03-637), Stockholm, Sweden.

**TABLE 1:** Characteristics of the commuter cyclists, their trips and cycling environments.

	**Age years**	**Height cm**	**Weight kg**	**BMI kg⋅m^–2^**	**HR rest beats⋅min^–1^**	**Duration min**	**Distance km**	**Velocity km⋅h^–1^**	**Trips per year**	Cycling environ -ment*
**Males** (*n* = 10)	43.6 ± 4.1	185 ± 7	85.0 ± 12.7	24.7 ± 3.0	59.2 ± 7.8	29.1 ± 7.2	9.61 ± 2.20	20.1 ± 2.7	366 ± 146	1.10 ± 0.32
**Females** (*n* = 10)	44.0 ± 2.6	170 ± 5	65.8 ± 7.7	22.6 ± 2.5	59.8 ± 5.6	23.2 ± 5.0	6.51 ± 1.51	16.8 ± 1.9	370 ± 117	0.90 ± 0.57

Values are presented as mean ± SD. *Cycling environment: 0 = inner urban; 1 = inner urban-suburban; 2 = suburban.

### Study Design and Standardization

The present study included three different test occasions of continuous measurements of HR and V̇O_2_ while cycling; twice in the laboratory on an ergometer cycle including both submaximal and maximal exercise, and once during each participant’s normal daily cycle commute. The reason for repeating the cycle ergometer exercise was to familiarize the participants with the procedure during a first occasion. Therefore, the values obtained from the second cycle ergometer occasion have been used as references and compared to the field occasion. However, due to technical problems with the MMS, which had to be solved and the equipment re-evaluated, 9 to 12 months elapsed before 14 of the field tests took place. For that reason, these participants performed one more occasion of cycle ergometer exercise in the laboratory. Thus, in these cases, the values from the extra laboratory test were used as references values. The mean time between the reference laboratory occasion and the field occasion was 15 ± 10 days (mean ± SD). Two trained investigators carried out the laboratory tests, each participant having the same investigator for each test. Three investigators completed the field tests, the same investigator always being in charge of the metabolic measurements.

Prior to all test occasions, the participants were instructed to follow the same standard procedures. These were: (1) not to engage in any vigorous exercise for 24 h beforehand, (2) not to cycle to the laboratory, (3) to refrain from eating, drinking, smoking, and taking snuff for at least 1 h before the test, (4) not to eat a large meal at least 3 h before the test, (5) to avoid stress, and (6) to cancel the test if they had fever, an infection or a cold.

### Procedure

#### Laboratory Tests

##### Rest measurements

On arrival at the laboratory, measurements of body weight and height were conducted. Thereafter, a resting HR measurement followed while the participants rested quietly in supine position on a treatment table during 10 min. The values from the last 5 min were used for determining the resting HR.

##### Cycle ergometer exercise

The submaximal cycle ergometer exercise was performed at 50, 100, and 150 watt (W) for the women, and 100, 150, and 200 W for the men. A cadence of 50 revolutions per minute (rpm) was used in accordance with Åstrand (1952, p. 19). At each work rate, the participants cycled until steady state HR was attained (approximately 6 min), after which the resistance was increased. The third work rate was increased to only 125 W or 175 W for women and men, respectively, if, after the second work rate, the participant’s HR was higher than 150 beats⋅min^–1^ and their rated perceived exertion (RPE) exceeded 15 (Borg, 1998, p. 30). Between the second and third work rates, the participants continued cycling for 1 min at a self-chosen low cadence with a resistance of 5 Newton (N). The participants were then asked to resume the cadence of 50 rpm while the investigator slowly increased the workload until, after 1 min, the third work rate was reached (resistance was increased to 50 W during the first 15 s, to 100 W the next 15 s and successively to the required workload during the last 30 s). After the submaximal exercise, and before the maximal test, the participants continued cycling for 2 min at a self-chosen low cadence at a resistance of 5 N.

The maximal test was performed with a cycle cadence of 80 rpm. During the first 3 min, the workloads were 60, 100, and 120 or 140 W for 1 min each. The latter alternatives depended on which third submaximal work rate the participants had performed. Thus, 120 W was chosen if the third submaximal work rate had been 125 or 175 W for women and men, respectively, while 140 W was used if it had been 150 or 200 W for women and men, respectively. After these first 3 min, the resistance increased by 20 W every minute until voluntary exhaustion occurred and ended the test. To ensure that the maximal tests achieved their purposes, at least two of the following three criteria were met by each participant: (1) a plateau in V̇O_2_ despite increasing exercise intensity (defined as a V̇O_2_ increment of <150 ml), (2) a RER of ≥1.1, and (3) a rating of RPE of ≥17 on the Borg scale (Borg, 1970; Howley et al., 1995; Midgley et al., 2007). To assess RPE, participants were asked to rate their perceived exertion for legs and breathing, separately, at the end of each submaximal work rate as well as immediately after finishing the maximal test.

#### Field Tests

##### Cycle commute

The participants rode their own bicycles either to or from their work-place choosing themselves which direction and time was most convenient. Information about the participants’ bicycles, such as weight and number of gears, is given in Schantz et al. (2020). All field tests were carried out between June and November. 18 of the participants were tested in the morning rush hours and the remaining two were tested in the evening rush hours. The commuting trips took place in the inner urban and suburban areas of Greater Stockholm, Sweden. A detailed description of these areas can be found in Wahlgren and Schantz (2011). The participants were met at the designated address by one of the investigators who transported the measurement equipment by car. Immediately before the cycling commute, the MMS was placed in a custom-made backpack on the participant. A GPS was also placed in the backpack to track the route. This was used for comparisons with the routes drawn on maps by the participants, and all were asked afterward to confirm which route they had taken. The starting time of the commuting trip was synchronized with a second investigator waiting at the destination. On arrival at the destination, the total trip time was noted by the second investigator. The participants were then asked to rate RPE of the overall trip and to state how many stops they made at traffic lights as well as other stops, and to mark them on the maps with their routes. Both the RPE ratings and the number of stops during the commutes are reported in Schantz et al. (2020).

The average ambient conditions (temperature, relative humidity, and wind speed) during the cycle commuting trips were obtained from the website of Stockholm-Uppsala Air Quality Management Association (2009). These are shown in Table 2.

**TABLE 2:** Ambient conditions during cycling commuting.

	**Temperature °C**	**Relative humidity %**	**Wind speed m⋅s^–1^**
**Males** (*n* = 10)	10 ± 4	77 ± 12	4.0 ± 2.0
**Females** (*n* = 10)	12 ± 4	71 ± 22	4.4 ± 1.9

*Values are presented as mean ± SD.*

### Quality Controls of Metabolic Systems

Methodological measurement premises will be stated here. A basic methodological prerequisite for this study is that the measurement systems in the laboratory and in the field cycle commuting provide the same and correct values at a given level of V̇O_2_, and that they prevail during the whole measurement period in the field.

In the laboratory, a SMS was used, while a MMS was used in the field. The brand of MMS used was previously compared with the Douglas bag system, and found to be valid during a variety of work rates during ergometer cycling in indoor conditions (Rosdahl et al., 2010). Further validity studies of the brand of the MMS were also undertaken during sustained physical work in outdoor conditions with relatively low temperatures and high humidity with results showing valid and stable measurements over time (up to about 45 min). Furthermore, varying severe external wind conditions were shown to not affect the results (Salier Eriksson et al., 2012). Comparisons of V̇O_2_ measurements between the SMS and the MMS were also undertaken in the laboratory (Schantz et al., 2018). At a range of four work rates demanding V̇O_2_ levels between 1.5–3.8 L⋅min^–1^, the relative differences were between −0.2–3.0% (n.s.). Thus, if there are any differences between the two metabolic systems, they are of low magnitude.

For technical and logistic reasons, the laboratory and field measurements took almost 2 years to complete. Therefore, to control for stability of both the SMS and MMS, we used a metabolic simulator (Vacumed 17056, Ventura, CA, United States), and checked a range of V̇O_2_ values between 1–4 L⋅min^–1^. There were no systematic difference between these systems during that time period (Schantz et al., 2018). Further checks related to the field measurements aimed to disclose any measurement drifts that could have resulted in a systematic non-stability. The results of these checks are reported in detail in Schantz et al. (2018), and led to the conclusion that there were no signs of a systematic drift in the V̇O_2_ measurements during the field studies. Finally, checks of the calibration factors before and after the field measurements (*n* = 15) indicated slightly lower oxygen values (2.1 ± 2.8%; *P* < 0.01) due to a drift in calibrations factors. The time period between the pre and post calibration is, however, a longer period than the actual active commuting (Schantz et al., 2018). Overall, the accumulated evidence from the quality control studies indicates that the deviations in the MMS from the correct values appear to be, at most, of a small magnitude of just some percent.

### Analytical Approach of HR Methods

The cycle ergometer exercise from the reference test in the laboratory was used to establish HR-V̇O_2_ relationships for estimating V̇O_2_ in the field. Paired HR and V̇O_2_ values from the last minute of each submaximal work rate were averaged and used for these analyses. Values from the maximal test were also used, and determined by averaging the highest consecutive paired values of HR and V̇O_2_ during 1 min. Based on these HR and V̇O_2_ values, two models of HR-V̇O_2_ relationships were created and calculated through linear regression equations for each individual. Model 1 included only the HR and V̇O_2_ values from the three submaximal work rates, whereas model 2 included both the submaximal and maximal HR and V̇O_2_ values.

**FIGURE 1:**
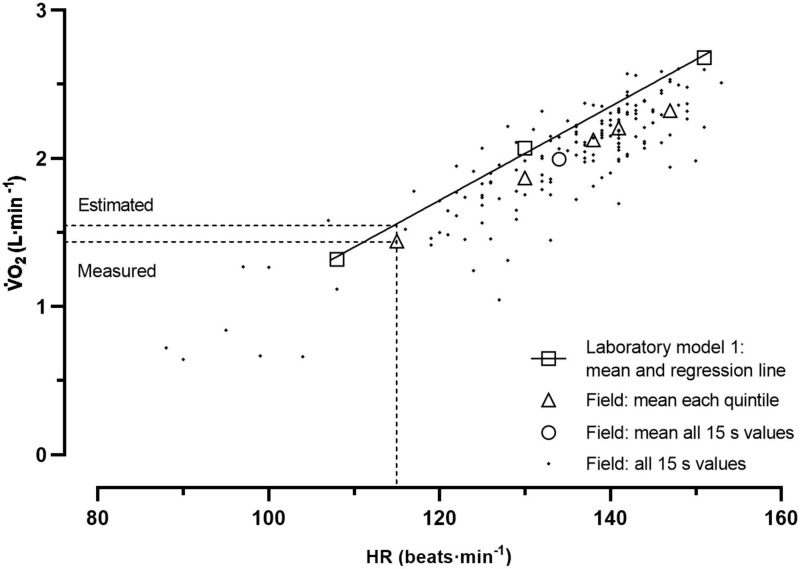
An individual example of the heart rate (HR) and oxygen uptake (V̇O_2_) measurements during the cycle commuting in field as well as the estimated V̇O_2_ based on the HR-V̇O_2_ relationship from the submaximal ergometer cycling (model 1) in the laboratory.

To capture possible nuances along the intensity spectrums of the cycling commutes, all field HR values were individually sorted from lowest to highest (while the measured V̇O_2_ remained attached to its corresponding HR). The sorted data was divided into five quintiles (0–20, 21–40, 41–60, 61–80, and 81–100%) of peak HR during the commute, and were first checked for normality of distribution. The individual measured HR and V̇O_2_ averages were thereafter calculated for each of the five quintiles, as well as for the whole commute. The six average HR values were then used in the laboratory established HR-V̇O_2_ regressions equations to estimate field V̇O_2_ for each individual. The estimations obtained at the six HR positions were thereafter compared with the corresponding measured V̇O_2_ values for the two models separately. An illustration of this analytical approach is shown in Figure 1.

### Statistical Analyses

The statistical analyses were performed using the Statistical Package for the Social Sciences (SPSS, 26.0, Chicago, IL, United States). Figures were created in GraphPad Prism 8.0 software package (GraphPad Software Inc., San Diego, CA, United States). Values are presented as mean ± SD unless otherwise stated. *P*-values less than 0.05 were considered as statistically significant. Although the same measured V̇O_2_ values were used twice (in models 1 and 2) for comparisons, no Bonferroni correction was applied, so as not to diminish the sensitivity of these analyses.

Student’s one-sample *t*-test was used for evaluating absolute and relative differences between the estimated and measured V̇O_2_ at the six positions of HR (five quintiles and the mean value of the whole commute). Confidence intervals (CI) of 95% were calculated for these differences. The agreement between the estimated and measured V̇O_2_ was also graphically displayed in Bland-Altman plots with 95% limits of agreement in both absolute and relative terms (Bland and Altman, 1986).

Power calculations were undertaken to evaluate the number of participants required to detect a relative difference of 10% between the estimated and measured V̇O_2_ with a statistical power of 0.90. Observed standard deviations, when investigating the reproducibility of the HR method based on cycle commuters (Schantz et al., 2019b), were used in an online power calculator (HyLown Consulting LLC, 2021). The results indicated that ten participants would be more than sufficient, and therefore it was appropriate to perform separate analyses for the sexes.

## Results

### Cycle Ergometer Work Rates in the Laboratory

Three work rates of submaximal ergometer cycling in the laboratory were used to create the HR-V̇O_2_ regression equations for both models 1 and 2. They induced mean levels of HR ranging between 99–138 beats⋅min^–1^ for the males and 97–146 beats⋅min^–1^ for the females (Table 3). The corresponding maximal values, which were used only for model 2, were 174 and 176 beats⋅min^–1^ for the males and females, respectively. Further descriptive aspects (%HRR, %HRmax, V̇O_2_, %V̇O_2_max, and RPE) of the work rates used are given in Table 3.

**TABLE 3:** Heart rate (HR), oxygen uptake (V̇O_2_) and rated perceived exertion (RPE) of cycle ergometer work rates in the laboratory.

	**Males (*n* = 10)**				**Females (*n* = 10)**			
**Work rate**	**100 ± 0 W**	**150 ± 0 W**	**193 ± 12 W**	**Maximal**	**50 ± 0 W**	**100 ± 0 W**	**140 ± 13 W**	**Maximal**
**HR** (beats⋅min^–1^)	99 ± 8	118 ± 9	138 ± 11	174 ± 7	97 ± 8	122 ± 9	146 ± 9	176 ± 11
**%HRmax**	56.9 ± 2.8	68.0 ± 2.8	79.1 ± 3.6	100.0 ± 0.0	55.1 ± 2.9	69.5 ± 3.1	82.9 ± 4.2	100.0 ± 0.0
**%HRR**	34.5 ± 3.5	51.5 ± 4.1	68.4 ± 5.4	100.0 ± 0.0	31.8 ± 4.1	53.6 ± 4.8	73.9 ± 6.5	100.0 ± 0.0
**V̇O_2_** (L⋅min^–1^)	1.47 ± 0.11	2.11 ± 0.10	2.67 ± 0.18	4.02 ± 0.53	0.83 ± 0.08	1.42 ± 0.11	1.92 ± 0.17	2.65 ± 0.37
**%V̇O_2_max**	37.0 ± 3.8	53.1 ± 5.4	67.2 ± 6.8	100.0 ± 0.0	31.6 ± 2.8	53.9 ± 5.0	73.1 ± 7.5	100.0 ± 0.0
**RPE, legs**	10.3 ± 0.9	12.1 ± 1.5	14.2 ± 1.7	18.3 ± 1.2	9.3 ± 1.8	12.6 ± 1.5	14.7 ± 1.4	17.7 ± 1.5
**RPE, breathing**	10.5 ± 1.4	12.8 ± 1.8	14.2 ± 2.1	17.8 ± 1.4	9.1 ± 2.0	12.5 ± 1.0	14.5 ± 0.7	18.3 ± 1.5

*Values are presented as mean ± SD.*

### Intensity Spectrum in Cycle Commuting

The range of the five means of HR quintiles during cycle commuting corresponded to 46–77% of V̇O_2_max for the males, whereas the mean of the whole trip was 65% (Table 4). The corresponding values for the females were 44–82 and 65%, respectively, (Table 5). The HR levels and the absolute values of the measured V̇O_2_ at these positions are given in Tables 4, 5.

**TABLE 4:** The heart rate (HR) spectrum during cycle commuting in field as well as the measured and estimated oxygen uptake (V̇O_2_) at these positions for males.

	**Values based on the mean HR in size ordered segments of quintiles (Q) of all single HR values during cycle commuting in field**					**Values based on the mean HR in field**
**Males** (*n* = 10)	**Q1**	**Q2**	**Q3**	**Q4**	**Q5**	**Mean**
**HR** (beats⋅min^–1^)	116 ± 13	132 ± 14	139 ± 13	144 ± 13	151 ± 12	136 ± 13
%HRmax	66.7 ± 7.5	75.7 ± 8.7	79.7 ± 8.7	82.5 ± 8.4	86.8 ± 8.2	78.3 ± 8.2
%HRR	49.5 ± 11.3	63.3 ± 13.0	69.3 ± 13.0	73.5 ± 12.6	80.0 ± 12.3	67.2 ± 12.2
**Measured V̇O_2_** (L⋅min^–1^)	1.84 ± 0.41	2.45 ± 0.56	2.70 ± 0.61	2.89 ± 0.62	3.11 ± 0.67	2.60 ± 0.55
%V̇O_2_max	46.0 ± 10.2	60.8 ± 11.0	67.1 ± 11.3	71.7 ± 11.3	77.2 ± 12.1	64.7 ± 10.7
**Estimated V̇O_2_ with model 1** (L⋅min^–1^)*	2.02 ± 0.50	2.52 ± 0.67	2.74 ± 0.70	2.89 ± 0.70	3.13 ± 0.71	2.66 ± 0.65
Absolute difference model 1 (L⋅min^–1^)	0.18 ± 0.37 (−0.09–0.44)	0.07 ± 0.32 (−0.15–0.30)	0.04 ± 0.25 (−0.14–0.22)	0.00 ± 0.24 (−0.17–0.18)	0.02 ± 0.24 (−0.16–0.19)	0.06 ± 0.27 (−0.13–0.25)
*P*-values	0.164	0.479	0.632	0.973	0.837	0.495
Relative difference model 1 (%)	11.2 ± 20.2 (−3.2–25.7)	3.4 ± 13.1 (−5.9–12.8)	1.6 ± 9.3 (−5.1–8.2)	0.2 ± 8.5 (−5.8–6.3)	0.9 ± 8.3 (−5.1–6.8)	2.5 ± 10.4 (−4.9–9.9)
*P*-values	0.112	0.428	0.604	0.927	0.755	0.464
**Estimated V̇O_2_ with model 2** (L⋅min^–1^)**	2.04 ± 0.55	2.59 ± 0.77	2.82 ± 0.81	2.98 ± 0.80	3.23 ± 0.82	2.73 ± 0.74
Absolute difference model 2 (L⋅min^–1^)	0.20 ± 0.40 (−0.09–0.48)	0.14 ± 0.38 (−0.13–0.41)	0.12 ± 0.33 (−0.12–0.35)	0.09 ± 0.32 (−0.14–0.32)	0.12 ± 0.30 (−0.09–0.34)	0.13 ± 0.33 (−0.10–0.37)
*P*-values	0.150	0.277	0.280	0.386	0.230	0.235
Relative difference model 2 (%)	11.8 ± 20.5 (−2.9–26.5)	5.5 ± 14.3 (−4.7–15.8)	4.0 ± 10.7 (−3.6–11.7)	3.0 ± 10.0 (−4.2–10.1)	3.9 ± 9.3 (−2.7–10.6)	4.8 ± 11.6 (−3.5–13.1)
*P*-values	0.102	0.251	0.263	0.373	0.211	0.218

Values are presented as mean ± SD and (95% CI). *Calculated from the HR-V̇O_2_ relationship in the laboratory, including three submaximal work rates of ergometer cycling. **Calculated from the HR-V̇O_2_ relationship in the laboratory, including three submaximal work rates and one maximal work rate of ergometer cycling.

**TABLE 5:** The heart rate (HR) spectrum during cycle commuting in field as well as the measured and estimated oxygen uptake (V̇O_2_) at these positions for females.

	**Values based on the mean HR in size ordered segments of quintiles (Q) of all single HR values during cycle commuting in field**					**Values based on the mean HR in field**
**Females** (*n* = 10)	**Q1**	**Q2**	**Q3**	**Q4**	**Q5**	**Mean**
**HR** (beats⋅min^–1^)	118 ± 10	132 ± 7	139 ± 6	145 ± 6	153 ± 7	138 ± 6
%HRmax	67.2 ± 5.6	75.4 ± 4.4	79.1 ± 3.9	82.4 ± 3.6	87.3 ± 3.4	78.4 ± 3.9
%HRR	50.3 ± 7.1	62.8 ± 5.4	68.5 ± 4.7	73.4 ± 4.5	80.8 ± 4.6	67.3 ± 4.7
**Measured V̇O_2_** (L⋅min^–1^)	1.15 ± 0.25	1.57 ± 0.21	1.77 ± 0.21	1.91 ± 0.21	2.14 ± 0.21	1.71 ± 0.20
%V̇O_2_max	43.7 ± 8.6	59.8 ± 10.6	67.5 ± 11.2	73.2 ± 12.8	81.6 ± 10.1	65.4 ± 10.0
**Estimated V̇O_2_ with model 1** (L⋅min^–1^)*	1.32 ± 0.25	1.64 ± 0.22	1.79 ± 0.22	1.92 ± 0.23	2.11 ± 0.24	1.76 ± 0.22
Absolute difference model 1 (L⋅min^–1^)	0.17 ± 0.24 (−0.00–0.34)	0.08 ± 0.17 (−0.04–0.20)	0.02 ± 0.16 (−0.09–0.14)	0.01 ± 0.19 (−0.13–0.14)	−0.03 ± 0.19 (−0.16–0.11)	0.05 ± 0.17 (−0.07–0.17)
*P*-values	0.053	0.174	0.662	0.903	0.640	0.390
Relative difference model 1 (%)	17.0 ± 21.5 (1.7–32.4)	5.6 ± 11.1 (−2.3–13.5)	1.7 ± 9.0 (−4.7–8.1)	0.8 ± 10.1 (−6.5–8.0)	−1.2 ± 8.8 (−7.5–5.2)	3.2 ± 10.4 (−4.2–10.7)
*P*-values	0.033	0.146	0.560	0.815	0.685	0.348
**Estimated V̇O_2_ with model 2** (L⋅min^–1^)**	1.31 ± 0.25	1.64 ± 0.21	1.79 ± 0.19	1.92 ± 0.19	2.12 ± 0.21	1.76 ± 0.20
Absolute difference model 2 (L⋅min^–1^)	0.16 ± 0.24 (−0.02–0.33)	0.07 ± 0.18 (−0.06–0.20)	0.02 ± 0.18 (−0.11–0.15)	0.01 ± 0.22 (−0.15–0.16)	−0.02 ± 0.20 (−0.17–0.12)	0.05 ± 0.19 (−0.09–0.18)
*P*-values	0.072	0.233	0.726	0.912	0.724	0.454
Relative difference model 2 (%)	15.8 ± 21.7 (0.3–31.4)	5.5 ± 12.2 (−3.3–14.2)	1.9 ± 10.6 (−5.7–9.5)	1.1 ± 11.9 (−7.3–9.6)	−0.7 ± 9.8 (−7.7–6.3)	3.4 ± 11.5 (−4.9–11.6)
*P*-values	0.047	0.190	0.586	0.768	0.823	0.381

Values are presented as mean ± SD and (95% CI). *Calculated from the HR-V̇O_2_ relationship in the laboratory, including three submaximal work rates of ergometer cycling. **Calculated from the HR-V̇O_2_ relationship in the laboratory, including three submaximal work rates and one maximal work rate of ergometer cycling.

### Comparison Between the Estimated and Measured VO_2_ During Cycle Commuting

**FIGURE 2:**
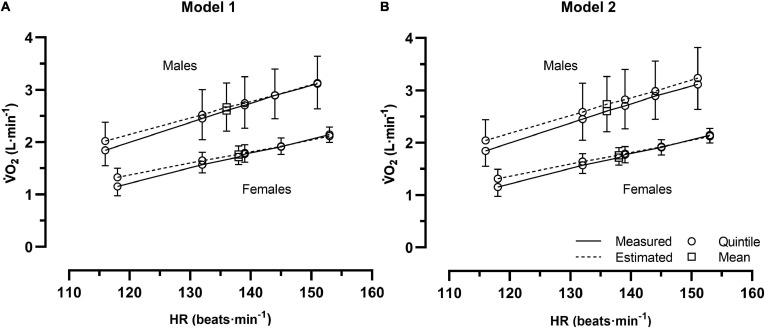
The measured and estimated oxygen uptake (V̇O_2_) with model 1 **(A)** and model 2 **(B)** between six positions of heart rate (HR) for males (*n* = 10) and females (*n* = 10), separately, during cycle commuting (mean plus the upper half, or minus the lower half of the 95% CI).

In the range of the six HR positions (five quintiles and a mean value) during cycle commuting, there were no significant absolute or relative differences between the estimated and measured V̇O_2_ for the males with either models 1 or 2. The ranges of the absolute V̇O_2_ differences were 0.00–0.18 L⋅min^–1^ (model 1) and 0.09–0.20 L⋅min^–1^ (model 2). The ranges of the relative V̇O_2_ differences were 0.2–11.2% (model 1) and 3.0–11.8% (model 2) (Table 4 and Figure 2).

For the females, no significant absolute V̇O_2_ differences were noted with either model 1 or model 2. The ranges of the absolute V̇O_2_ differences were −0.03–0.17 L⋅min^–1^ (model 1) and −0.02–0.16 L⋅min^–1^ (model 2). However, at the first HR quintile there was a tendency (*P* < 0.1) of 0.16–0.17 L⋅min^–1^ higher estimated V̇O_2_ in both models. These estimated V̇O_2_ values were in relative terms significantly higher than the measured; 17.0% (model 1) and 15.8% (model 2). The ranges of the other five relative differences with models 1 and 2 varied between −1.2–5.6% and −0.7–5.5%, respectively, (all n.s.) (Table 5 and Figure 2).

**FIGURE 3:**
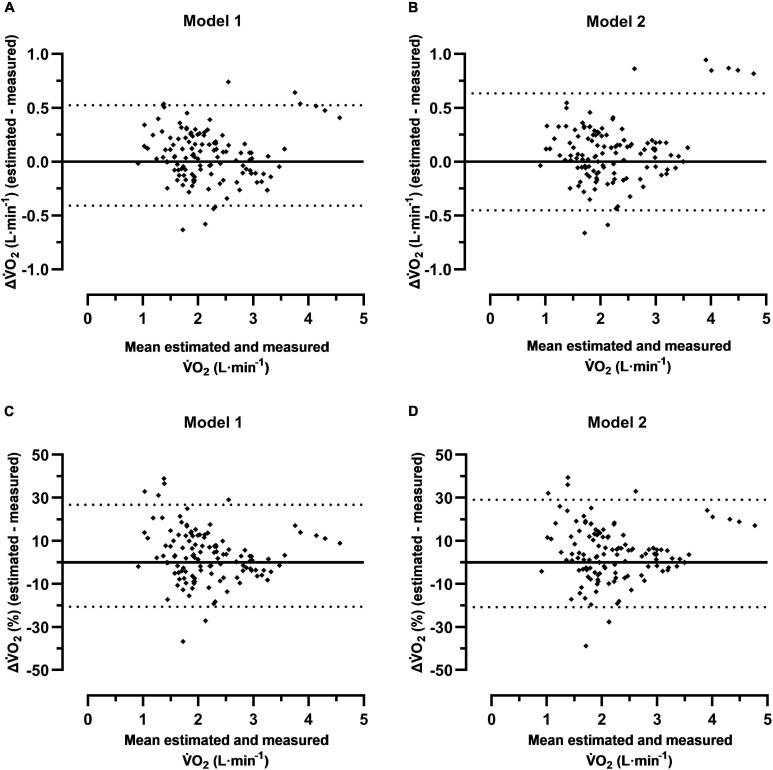
Individual levels of differences with 95% limits of agreement (dotted lines) between the estimated and measured oxygen uptake (V̇O_2_) at six positions of heart rate (HR) for males and females together (*n* = 20) during cycle commuting. The y-axes show the absolute or the relative differences against the mean values of the estimated and measured V̇O_2_ on the *x*-axes for the two models; absolute differences with model 1 **(A)** and model 2 **(B)**; relative differences with model 1 **(C)** and model 2 **(D)**.

The individual levels of differences between the estimated and measured V̇O_2_, including all participants, show that there are rather large individual variations in both models (Figure 3). The absolute 95% limits of agreement varied between −0.41–0.52 L⋅min^–1^ (model 1) and −0.45–0.63 L⋅min^–1^ (model 2), and the relative ranges were −20.6–26.8% (model 1) and −20.9–29.0% (model 2).

## Discussion

This is, to our knowledge, the first time that the validity of laboratory measured HR-V̇O_2_ relationships has been evaluated as a part of the HR method for estimating spectrums of oxygen uptake in field exercise. For this purpose, we applied the methodology on cycle commuting with habitual male and female commuter cyclists. The participants performed submaximal and maximal work rates of ergometer cycling in the laboratory, which were used to establish two models of linear HR-V̇O_2_ regression equations for estimating V̇O_2_ at six HR positions (five quintiles and a mean value) based on the cycle commutes.

The main finding was that no significant differences were noted between the estimated and measured V̇O_2_ for the males with both models of HR methods (Table 4). For the females, the estimated V̇O_2_ levels at the first HR quintile were, in absolute terms, not significantly different from the measured V̇O_2_ with both models, whereas the corresponding relative differences were significant (15.8–17.0%; Table 5). No other significant differences were noted at the five higher HR levels. The overall judgment was therefore that the two models of HR methods are valid at a group level for estimating V̇O_2_ along exercise intensity spectrums between approximately 45–80% of V̇O_2_max during cycle commuting in a metropolitan setting. However, rather large individual variations were indicated by the 95% limits of agreement of all individual levels of differences between the estimated and measured V̇O_2_ (model 1: −20.6–26.8%; Figure 3C and model 2: −20.9–29.0%; Figure 3D).

Possible reasons for the deviations between the estimated and measured V̇O_2_, at both the individual and group level, need to be interpreted from various perspectives, such as distinguishing between technical and exercise related aspects that may have influenced these results. On the technical side, two different metabolic measurement systems were used in laboratory and field conditions. Hence, a series of methodological validity studies and checks were undertaken prior to and during the present study, and they secured concordance between the two measurement systems and conditions (Rosdahl et al., 2010; Salier Eriksson et al., 2012; Schantz et al., 2018; see Methods).

Another important aspect to consider is the reproducibility of the HR method when estimating V̇O_2_. This is because the day-to-day variability in the HR-V̇O_2_ relationship can explain parts of the individual variations observed between the estimated and measured V̇O_2_. Therefore, it is of relevance to compare the present relative differences with test and retest values at a matching intensity level. For example, if studying the third HR quintile (Q3), the mean difference between the estimated and measured V̇O_2_ when using model 1 was 1.6 ± 9.3% for the males (Table 4) and 1.7 ± 9.0% for the females (Table 5). When comparing against the test and retest value at the matching HR position, the difference for males and females together (*n* = 19) was 2.7 ± 6.5% with model 1 (Schantz et al., 2019b). The corresponding finding was noted with model 2. Thus, a great part of the individual variations between the estimated and measured V̇O_2_ can be explained by the day-to-day variability in the HR-V̇O_2_ relationships. In order to minimize this form of variability, we suggest, according to our unpublished results, that the HR-V̇O_2_ regression equations are based on five rather than three submaximal measurement points.

For interpreting the results, the different measurement ranges of the laboratory tests and cycle commutes are also important to comment on. Ideally there should be a complete overlap in HR and V̇O_2_ levels between the two measurement conditions. This is since the HR-V̇O_2_ relationship, although being linear for a great part of the spectrum between rest and maximal intensity, can, on an individual level, diverge from the linearity at both very low and high intensity levels (Booyens and Hervey, 1960; Davies, 1968; Luke et al., 1997; Achten and Jeukendrup, 2003). When comparing the %V̇O_2_max ranges for the submaximal exercise in the laboratory (males: 37–67% and females 32–73%; Table 3) with the corresponding ranges in the field (males: 46–77%; Table 4 and females: 44–82%; Table 5), it is notable that these ranges overlap each other to a great extent.

With model 2, the addition of a maximal measurement point leads to a complete overlap of the two different ranges. Interestingly, this does not lead to more accurate estimations of V̇O_2_. On the contrary, slightly higher mean V̇O_2_ levels were observed with model 2 than compared to model 1 for the males (Table 4 and Figure 2). Although this has not been statistically evaluated, it is in line with previous research (Davies, 1968; Åstrand et al., 2003, p. 285) that has shown that V̇O_2_ in some individuals increases proportionally more than HR in the measurement region close to V̇O_2_max. Furthermore, as shown in Olsson et al. (2020), there is not a stabilizing effect on the individual variations when a maximal measurement point is included in the HR-V̇O_2_ relationship (model 2) based on ergometer cycling. Overall, this indicates that model 2 does not add any value compared to model 1 when applied to, at least, cycle commuting.

The only significant differences between the estimated and measured V̇O_2_ were at the first HR quintile for the females (Table 5). Although not significant, a similar degree of difference appears for the males (Table 4). Why do such differences not protrude at higher intensity levels? At least two possible explanations exist. If there is an effect of stress on HR by the traffic environment, then it may have a greater potential to affect the HR at lower levels (Carroll et al., 2009; Hahad et al., 2019). Another potential explanation is that the oxygen pulse at a given V̇O_2_ is altered in non-steady state conditions, and in conjunction with lower HR levels. Support for this comes from experimental laboratory conditions with higher HR at given V̇O_2_ levels during non-steady state compared to steady state conditions (Gilbert and Auchincloss, 1971). This would be valuable to study further.

The present findings should be viewed in relation to the durations of the cycle commutes of about 25 min (Table 1) and the relative mean exercise intensity of approximately 65% of V̇O_2_max (Tables 4, 5). Given this, it is possible that a certain drift in HR and/or V̇O_2_ levels may have occurred in some individuals (Ekelund, 1967; Coyle and Gonzalez-Alonso, 2001; Achten and Jeukendrup, 2003; Zuccarelli et al., 2018). In a study by Zuccarelli et al. (2018), it was observed that the HR drift occurred at a lower intensity level during constant exercise than compared to V̇O_2_. In such cases, the HR method needs to compensate for that. The overall high level of agreement between the estimated and measured V̇O_2_ in the present study contradicts this disproportionate drift being a problem for this group of participants under the conditions studied. We recommend, however, that this method is also checked for other groups of participants, for example less experienced cyclists. Furthermore it is of value to also validate the method in other climatologic conditions. This is since it is expected that HR drifts occur to a greater extent at higher temperatures than in the present ambient temperatures (around 10–12°C) studied (cf. Achten and Jeukendrup, 2003).

When comparing the steady state conditions in laboratory with the varying intensities during the field measurements, we have noted a phenomenon that affects the variability in the oxygen pulse (V̇O_2_/HR) along the oxygen uptake spectrums of the cycle commuting. It deserves a comment, and it is illustrated in Figure 1. It represents a typical example from one individual of a spreading of all pairs of HR and V̇O_2_ values based on the 15 s measurement periods in field. In each HR value one can note a great spreading in V̇O_2_ levels. What can explain that? Through visual inspections of the individual patterns of alterations in levels of HR and V̇O_2_ in each 15 s period (not shown), it can be noted that the changes in V̇O_2_, with both upward and downward alterations of greater magnitude, often precede those observed in HR. Thus, a consequence of this on the oxygen pulse, as compared to steady state conditions, is that when the V̇O_2_ increases and the HR lags, the oxygen pulse at a given V̇O_2_ will be higher than at steady state, while the opposite occurs when V̇O_2_ decreases and the HR lags, i.e., lower oxygen pulse than at steady state. This may be balanced when averaging all the alterations in V̇O_2_ during a cycle commuting trip. Still, it can, at least partly, explain the rather great spreading of V̇O_2_ values at given levels of HR for the 15 s periods.

This leads us to the issue of how to characterize exercise intensities during free-living physical activities. As stated in the introduction, this is of importance for understanding the relation between human movement, health and performance outcomes. In the present study, we have been able to show rather large intensity variations around the mean values in field. Thus, it appears problematic to characterize this form of physical activity by only presenting mean values, since it might hide important information. We therefore suggest that future studies search for more elaborate ways of describing intensity variations, and to those endeavors include intensity variations per unit of time.

The general importance of this can be viewed from the perspective of that, although health outcomes are coupled to a physical activity such as cycle commuting (e.g., Andersen et al., 2000; Hu et al., 2003, 2007), very little data is available concerning the physical activity bases for such health outcomes in terms of exercise intensities, trip durations and frequencies of exercise (Stigell and Schantz, 2015; Schantz, 2017; Schantz et al., 2020). This needs to be surveyed in local contexts, since cycling culture, demography, infrastructure and topography might induce qualitative differences in the commuter cycling. The present findings open up for such studies on a valid basis, as well as simpler and broader scales than previous methods. Furthermore, it enables the method to be used for health education and promotion, and indeed given the role cycling can play, not only for the individual and for public health, but also for the planetary health (e.g., Johansson et al., 2017; Brand et al., 2021), this represents a very welcome methodological advancement.

A final comment relates to that a limitation of this study is that the HR methods are based on only three submaximal work rates, which might have led to the rather high individual variability observed. Furthermore, the number of participants was rather low. On the other hand, both males and females were studied. A great strength is that validated methods have been used to evaluate HR methods that are applied in contexts of exercise intensity spectrums and field conditions.

In conclusion, the present study indicates that HR methods are valid on a group level for estimating V̇O_2_ in cycle commuting characterized by relatively wide spectrums of exercise intensities. Further studies need to investigate whether the same applies to other forms of field exercises.

## Data Availability Statement

The raw data supporting the conclusions of this article will be made available by the authors, without undue reservation.

## Ethics Statement

The studies involving human participants were reviewed and approved by the Ethics Committee North of the Karolinska Institute at the Karolinska Hospital (Dnr 03-637), Stockholm, Sweden. The patients/participants provided their written informed consent to participate in this study.

## Author Contributions

PS conceived the overall aim of the study and designed it. JSE, HR, and PS were responsible for collecting and analyzing the data. KO was responsible for analyzing data and illustrating them. HR and PS were responsible for the quality of the measurement devices in the laboratory and in the field. JSE drafted the first version of the introduction and method sections. PS and KO drafted the remainder of the manuscript. All authors read, commented, and accepted the final manuscript.

## Conflict of Interest

The authors declare that the research was conducted in the absence of any commercial or financial relationships that could be construed as a potential conflict of interest.
